# Mitochondrial DNA haplogroups and risk of attention deficit and hyperactivity disorder in European Americans

**DOI:** 10.1038/s41398-020-01064-1

**Published:** 2020-11-02

**Authors:** Xiao Chang, Yichuan Liu, Frank Mentch, Joseph Glessner, Huiqi Qu, Kenny Nguyen, Patrick M. A. Sleiman, Hakon Hakonarson

**Affiliations:** 1 Center for Applied Genomics, The Children’s Hospital of Philadelphia, Philadelphia, PA USA; 2 Department of Pediatrics, The Perelman School of Medicine, University of Pennsylvania, Philadelphia, PA USA; 3 Division of Human Genetics, Department of Pediatrics, The Perelman School of Medicine, University of Pennsylvania, Philadelphia, PA USA

**Keywords:** ADHD, Genetics

## Abstract

Although mitochondrial dysfunction has been implicated in the pathophysiology of attention deficit and hyperactivity disorder ADHD, the role of mitochondrial DNA (mtDNA) has not been extensively investigated. To determine whether mtDNA haplogroups influence risk of ADHD, we performed a case-control study comprising 2076 ADHD cases and 5078 healthy controls, all of whom were European decedents recruited from The Children’s Hospital of Philadelphia (CHOP). Associations between eight major European mtDNA Haplogroups and ADHD risk were assessed in three independent European cohorts. Meta-analysis of the three studies indicated that mtDNA haplogroups K (odds ratio = 0.69, *P* = 2.24 × 10^−4^, *P*_corrected_ = 1.79 × 10^−3^) and U (odds ratio = 0.77, *P* = 8.88 × 10^−4^, *P*_corrected_ = 7.11 × 10^−3^) were significantly associated with reduced risk of ADHD. In contrast, haplogroup HHV* (odds ratio = 1.18, *P* = 2.32 × 10^−3^, *P*_corrected_ = 0.019) was significantly associated with increased risk of ADHD. Our results provide novel insight into the genetic basis of ADHD, implicating mitochondrial mechanisms in the pathophysiology of this relatively common psychiatric disorder.

## Introduction

Attention-deficit hyperactivity disorder (ADHD) is a common neurodevelopmental disorder in children and adolescents, characterized by symptoms of hyperactivity, impulsivity, and inattention. It has been recognized as a highly heritable disorder with a twin-estimated heritability around 80%^1^. Genetic advances in ADHD revealed a polygenic genetic architecture with multiple common and rare genetic factors involved^1^. Recent genome-wide association studies (GWAS) have identified common variants at 12 loci that are significantly associated ADHD risk through analyzing 20,183 ADHD cases and 35,191 controls^2^. Rare copy number variations (CNVs) and single-nucleotide variants (SNVs) have also been reported in multiple ADHD studies^3–9^. However, most genetic studies of ADHD focused on nuclear DNA (nDNA), and detected susceptibility genes of ADHD so far only account for a small fraction of the genetic contribution to the disease. Mitochondrial DNA (mtDNA) therefore emerges as an appealing candidate for the heritability of ADHD.

Mitochondria are best known for their pivotal role in energy production but are also involved in other cellular processes, such as amino-acid, lipid and steroid metabolism, and regulation of apoptosis^10^. Human mitochondria possess a small circular genome, which sequentially accumulates genetic variants via maternal inheritance. Hence, human mitochondria genomes are classified into several mtDNA haplogroups according to accumulated mtDNA variants during ancient migrations of human populations^11^. Previous studies indicate that mtDNA haplogroups display diverse metabolic capacities, and play an important role in multiple diseases and traits, such as cancers, neurodegenerative diseases, and longevity^12–16^. Recently, cumulative evidence suggested that mitochondrial dysfunction might also lead to a disruption of normal neuronal plasticity and promote the development of psychiatric disorders^10,17^, implying a potential impact of mtDNA haplogroups in the risk of neuropsychiatric disorders, such as ADHD. However, to the best of our knowledge, the association between mtDNA haplogroups and ADHD has been only evaluated in a small Korean population of 150 cases and 322 controls^18^. Thus, the genetic relationship between mtDNA haplogroups and ADHD has not yet been studied in European populations.

In this study, we investigated the mtDNA haplogroup distribution in three independent European cohorts of patients with ADHD and compared them with healthy control individuals, aiming to identify potential mtDNA haplogroups conferring risk to ADHD in European populations.

## Methods

### Participants

This case-control study included three independent ADHD cohorts. Blood-derived DNA from participants from the three cohorts were genotyped at the Center for Applied Genomics (CAG), at The Children’s Hospital of Philadelphia (CHOP), using the Illumina HumanHap550 (HH550, cohort 1), HumanHap610 (HH610, cohort 2), and Infinium Global Screening (GSA) SNP arrays (GSA, cohort 3), respectively.

Cases genotyped of the HH550 chip were recruited from the pediatric and behavioral health clinics at CHOP, capturing the greater Philadelphia area and included children (age 6–18 y/o) diagnosed with ADHD following the K-SADS (Schedule for Affective Disorders and Schizophrenia for School-Age Children; Epidemiologic Version) interview. Exclusion criteria were prematurity (<36 weeks), intellectual disability, major medical and neurological disorders, pervasive developmental disorder, psychoses, and major mood disorders as described previously^4,19^. Controls for the HH550 cohort were recruited by CAG through the CHOP Health Care Network including four primary care clinics and several group practices and outpatient practices that performed well-child visits. Healthy children aged 6–18 years old, with no serious underlying medical disorder, including but not limited to neurodevelopmental disorders, cancer, chromosomal abnormalities, and known metabolic or genetic disorders were included^4,19^.

Individuals genotyped on the HH610 and GSA arrays were selected from the biorepository at the CAG, which collected over 100,000 unique samples recruited from CHOP from subjects linked to their electronic health records (EHRs). Cases and controls were selected based on diagnostic and medication information from EHRs. Inclusion criteria for ADHD cases included (1) ages above four years old; (2) two or more diagnosis of ICD9 codes for ADHD (314.1–314.9) in two separate in-person visits and more than two prescriptions of ADHD-related medications; or (3) three or more ICD9 codes for ADHD on separate calendar days. Exclusion criteria for ADHD cases were conditions related to brain anomalies or tumors, mental retardation, or psychiatric disorders. Controls were determined based on the following criteria (1) ages above four years old; (2) no diagnosis of ICD9 codes for ADHD or other psychiatric, neurological and related disorders; (3) no mention of the terms “ADHD”, “attention deficit”, “hyperkinesia”, “hyperkinesis”, “hyperkinetic”, or “hyperactivity”; (4) no ADHD related medication. The ADHD case and control selection method was further validated by manual chart review of 100 randomly selected cases (*N* = 50) and controls (*N* = 50), and yielded positive predictive values (PPVs) >96% for both cases and controls. All three groups were analyzed separately with their array and age/sex, race matched controls, and then meta-analyzed for the study as described in the results section.

Written informed consent was obtained from all participants by nursing and medical assistant study staff under the direction of CHOP clinicians. All data were analyzed anonymously and all clinical investigations were conducted according to the principles expressed in the Declaration of Helsinki. This study was approved by the Research Ethics Board of CHOP (CHOP IRB#: IRB 06-004886).

### Haplogroup assignment

mtDNA SNV markers interrogated by the HumanHap550, HumanHap610, and GSA SNP arrays were used in this analysis. There are 163 mtDNA markers included on the HumanHap550 SNP array, 138 mtDNA markers on the HumanHap610 SNP array, and 131 mtDNA markers on the GSA SNP array. The coordinates of mtDNA markers in the HumanHap550 and HumanHap610 SNP array were based on the AF347015.1 mitochondrial reference genome, which were converted to the revised Cambridge Reference Sequence (rCRS, GenBank NC012920). The mtDNA haplogroup of each participant was determined by HaploGrep 2^20^. Given ambiguities in precise haplogroup assignment, evolutionary related haplogroups B, F, H, HV, P, R, and V were classified as the super-haplogroup HHV*; haplogroups O and X were classified as the super-haplogroup OX. The accuracy, sensitivity, and specificity of mtDNA haplogroup assignment were verified using the three sets of SNVs in 11240 mitochondrial reference genomes with known haplogroups, downloaded from GenBank. Eight haplogroups common in European populations, including HHV*, I, J, K, OX, T, U, and W, were analyzed in this study (Supplementary Table S1).

### Statistical analysis

The association between each haplogroup and ADHD risk was tested in R (version 3.5.1) using logistic regression analyses. Gender and the first ten principal components were included as covariates. Principal component analysis (PCA) was performed using EIGENSTRAT to infer genetic ancestry of samples and detect population substructures^21^. Participants with European ancestry were selected by comparing with four reference European populations from 1000 Genome, including Utah Residents with Northern and Western European ancestry (CEU), Toscani in Italia (TSI), British in England and Scotland (GBR), and Iberian Population in Spain (IBS). PCA plots of the population structures are provided in Supplementary Fig. S1. Meta-analysis of summary statistics from the three cohorts was performed by PLINK (version 1.9)^22^. Fixed effects *P* values were reported. Bonferroni correction was used for correction of multiple testing. A corrected *P* < 0.05 was considered statistically significant. The kinship between pairs of subjects was detected by identity by descent (IBD) analysis, and one of the related individuals with proportional IBD > 0.25 were excluded.

## Results

The HH550 cohort comprising 764 European-American ADHD cases and 2031 ancestry-matched controls was used in the discovery analysis. Haplogroups including I, J, K, L, T, W, OX, and HHV* were tested, as they were mainly presented in European populations. In consistent with this notion, more than 97% of individuals in the HH550 cohort belonged to the eight common European haplogroups. In addition, the HHV* haplogroup encompassed about 50% of subjects in the HH550 cohort, which was consistent with previous mtDNA studies of European populations^23,24^. Association analyses indicated that haplogroup K was significantly less represented in patients with ADHD than healthy controls (odds ratio 0.64, *P* = 0.00443, *P*_corrected_ = 0.0354, Table 1), suggesting a protective role of haplogroup K in ADHD risk.

**Table 1:** Associations of eight haplogroups in three European cohorts.

Study	Haplogroup	*N*_case_	*N*_control_	*F*_case_	*F*_control_	OR (95% CI)	*P*	*P*_corrected_
HH550	OX	8	34	1.05%	1.67%	0.62 (0.29,1.35)	0.244	1
	W	17	31	2.23%	1.53%	1.47 (0.81,2.67)	0.268	1
	K	50	199	6.54%	9.80%	0.64 (0.47,0.89)	4.43 × 10^−3^	0.035
	U	94	292	12.30%	14.38%	0.84 (0.65,1.07)	0.197	1
	J	71	204	9.29%	10.04%	0.92 (0.69,1.22)	0.653	1
	T	74	184	9.69%	9.06%	1.08 (0.81,1.43)	0.653	1
	I	30	58	3.93%	2.86%	1.39 (0.89,2.18)	0.13	1
	HHV*	396	972	51.83%	47.86%	1.17 (0.99,1.38)	0.071	0.564
	UK	144	491	18.85%	24.18%	0.73 (0.59,0.9)	2.63 × 10^−3^	0.021
HH610	OX	7	32	1.08%	1.60%	0.67 (0.29,1.52)	0.349	1
	W	10	32	1.54%	1.60%	0.96 (0.47,1.97)	0.865	1
	K	43	193	6.61%	9.64%	0.66 (0.47,0.93)	0.011	0.084
	U	73	304	11.21%	15.18%	0.71 (0.54,0.93)	0.023	0.186
	J	73	197	11.21%	9.84%	1.16 (0.87,1.54)	0.269	1
	T	70	196	10.75%	9.79%	1.11 (0.83,1.48)	0.437	1
	I	17	61	2.61%	3.05%	0.85 (0.5,1.47)	0.684	1
	HHV*	337	940	51.77%	46.93%	1.21 (1.02,1.45)	0.086	0.69
	UK	116	497	17.82%	24.81%	0.66 (0.52,0.82)	3.08 × 10^−4^	2.46 × 10^−3^
GSA	OX	0	2	0.00%	0.19%	0 (0,Inf)	0.972	1
	W	0	0	0.00%	0.00%	NA	NA	NA
	K	46	98	6.96%	9.39%	0.72 (0.5,1.04)	0.098	0.785
	U	78	162	11.80%	15.52%	0.73 (0.55,0.97)	0.046	0.368
	J	55	88	8.32%	8.43%	0.99 (0.69,1.4)	0.64	1
	T	66	98	9.98%	9.39%	1.07 (0.77,1.49)	0.574	1
	I	19	32	2.87%	3.07%	0.94 (0.53,1.67)	0.739	1
	HHV*	350	500	52.95%	47.89%	1.22 (1.01,1.49)	0.035	0.278
	UK	124	260	18.76%	24.90%	0.7 (0.55,0.89)	5.85 × 10^−3^	0.047

Associations between eight mtDNA haplogroups and ADHD risk were also assessed in an independent HH610 cohort containing 651 cases and 2003 controls. A significant association between Haplogroup K and ADHD risk was observed (odds ratio 0.66, *P* = 0.0105, Table 1). Interestingly, haplogroup U, from which haplogroup K was derived, was also significantly associated with reduced risk of ADHD (odds ratio 0.71, *P* = 0.0233, Table 1). We therefore combined haplogroups U and K as a super-haplogroup UK, and detected a significant association after adjustment for multiple testing between the UK haplogroup and ADHD risk (odds ratio 0.66, *P* = 3.08 × 10^−4^, *P*_corrected_ = 2.46 × 10^−3^, Table 1).

The association between the super-haplogroup UK and ADHD risk was further replicated in another cohort GSA including 661 cases and 1044 controls (odds ratio 0.7, *P* = 5.85 × 10^−3^, Table 1). Meta-analysis combing the association results from three studies indicated that both haplogroup K and U yielded a *P* value that extends well beyond the significance threshold after adjustment for multiple testing (Table 2). In addition, haplogroup HHV*, the most frequent haplogroup in European populations, was significantly associated with increased risk of ADHD after meta-analysis (odds ratio 1.176, *P* = 2.32 × 10^−3^, *P*_corrected_ = 0.0185, Table 2).

**Table 2:** Meta-analysis of results from three European cohorts.

HaploGroup	OR	*P*	*P*_corrected_	*I*
OX	0.63	0.119	0.953	0
W	1.18	0.478	1	0
K	0.69	2.24 × 10^−4^	1.79 × 10^−3^	0
U	0.77	8.88 × 10^−4^	7.11 × 10^−3^	0
J	1.05	0.611	1	0
T	1.08	0.404	1	0
I	1.09	0.586	1	3.29
HHV*	1.18	2.32 × 10^−3^	0.019	0
UK	0.71	1.94 × 10^−7^	1.55 × 10^−6^	0

*I*: *I*^2^ heterogeneity index (0–100)

## Discussion

To our knowledge, this is the first large-scale study to investigate the role of mtDNA haplogroups in ADHD in European populations. Our findings indicate that haplogroups U and K are associated with reduced risk of ADHD in patients of European descent, while the super-haplogroup HHV* is associated with increased risk of ADHD. Our findings demonstrate that genetic variations of mtDNA are implicated in the development of ADHD.

Although associations between mtDNA haplogroups and ADHD have been reported in a recent study of Korean children^18^, these findings need to be further verified by studies of larger sample sizes and other ethnicity populations. In this study, the association between mtDNA haplogroups and ADHD was consistently observed in three independent European populations, adding robust evidence to support the role of mtDNA haplogroups in ADHD. Cases and controls of the HH550 cohort used here were previously investigated in CNV and GWAS studies of ADHD^4,19^, which successfully identified multiple genetic risk factors of ADHD. Cases and controls from HH610 and GSA cohorts were determined based on recurrent use of diagnostic codes and medication information from EHRs. The accuracy of this selection method was further validated with high accuracy by manual chart review. Overall, cases and controls were appropriately selected for this case-control study. European subjects were strictly selected based on their genetic ancestry inferred from genotyping array data. Association results were further adjusted by the first ten PCs calculated from the genotyping array data in order to reduce the possibility of spurious association caused by population stratification,

A protective role of haplogroups K and U was observed in this study. Although they are classified as two major haplogroups in European populations, haplogroup K was recognized as a subclade of haplogroup U, implying those two haplogroups may have similar mitochondrial activities and functions. Consistent with this, haplogroups K and U displayed an uncoupling effect leading to reduced adenosine triphosphate (ATP) production and energy generation^15,16^. Cybrids of haplogroups K and U also displayed lower inner mitochondrial membrane potential, mitochondrial protein synthesis, and cell growth capacity than haplogroup H^25^. Hence, the protective effect of haplogroup K and U could be partially explained by energy deficiency and/or reduced load of harmful reactions. In addition, members of haplogroups K and U represented higher brain pH than others^26^. Higher pH is correlated with a lower brain activity and confers protection against psychiatric disorders^27^. In fact, haplogroup K and U have also been implicated in multiple neurological and neurodegenerative disorders in Europeans, such as transient ischemic attack, ischemic stroke, neuroblastoma, Parkinson’s disease, and Alzheimer’s disease^23,24,28–31^.

Our study also revealed that the HHV* haplogroup, the most dominant haplogroup in European populations, is associated with increased risk of ADHD. In contrast to haplogroup K and U, haplogroup H was associated with higher ATP levels and oxidative phosphorylation OXPHOS capacities^25,32^. It has also been reported to increase the risk of Alzheimer’s disease and lower the age onset of Huntington disease presentation^29,32^.

This study has two limitations. First, the mtDNA haplogroups were deduced from genotyping arrays. It remains unclear which precise genetic variants are causal in ADHD. This limitation can be solved in future studies by sequencing the whole mtDNA genome in a large collection of ADHD cases and controls. Second, the three study cohorts were genotyped at the same location, and it is possible that unknown potential confounding factors might have influenced the results. Third, the results could be confounded by maternal ancestry, since human mtDNA is maternally inherited. However, this effect is not likely to be significant as it is much smaller for the homogeneous European population than for admixed populations.

## Conclusion

This study demonstrates that mtDNA haplogroups K and U confer protective effects against ADHD, while mtDNA haplogroup HHV* is associated with increased risk of ADHD, providing new insights into the genetic basis of ADHD. A better understanding of the role of mitochondria in the pathogenesis of ADHD might lead to new methods for the prevention and treatment of this common disease.

## Supplementary information

Supplementary files
