# Supplementary material for: Mitochondrial DNA haplogroups and risk of attention deficit and hyperactivity disorder in European Americans
Source: Transl Psychiatry. 2020 Nov 2;10:370. doi: 10.1038/s41398-020-01064-1 (PMC7608630; doi:10.1038/s41398-020-01064-1)

Table S1. Sensitivities and specificities of haplogroup predictions.

HaploGroup	HH550		HH610		GSA	
	**Sensitivity**	**Specificity**	**Sensitivity**	**Specificity**	**Sensitivity**	**Specificity**
OX	100%	98.5%	99.3%	100%	8.1%	64.7%
W	100%	98.5%	94.5%	98.4%	15.5%	100%
K	100%	100%	100%	100%	96.4%	99.5%
U	100%	100%	100%	100%	95.5%	99.7%
J	100%	100%	100%	100%	98.8%	98.8%
T	100%	100%	99.3%	100%	98.4%	100%
I	100%	100%	100%	99.2%	100%	97.8%
HHV*	97.8%	99.9%	98%	97.1%	94.7%	91.4%

Figure S1. Population structures of the study samples revealed by principal component analysis. Cases and controls are shown as black and grey dots respectively. Four reference European populations from 1000 Genome, including Utah Residents with Northern and Western European Ancestry (CEU), Toscani in Italia (TSI), British in England and Scotland (GBR), and Iberian Population in Spain (IBS) are shown as green, purple, blue and orange respectively.

A. Population structures of the HH550 cohort.

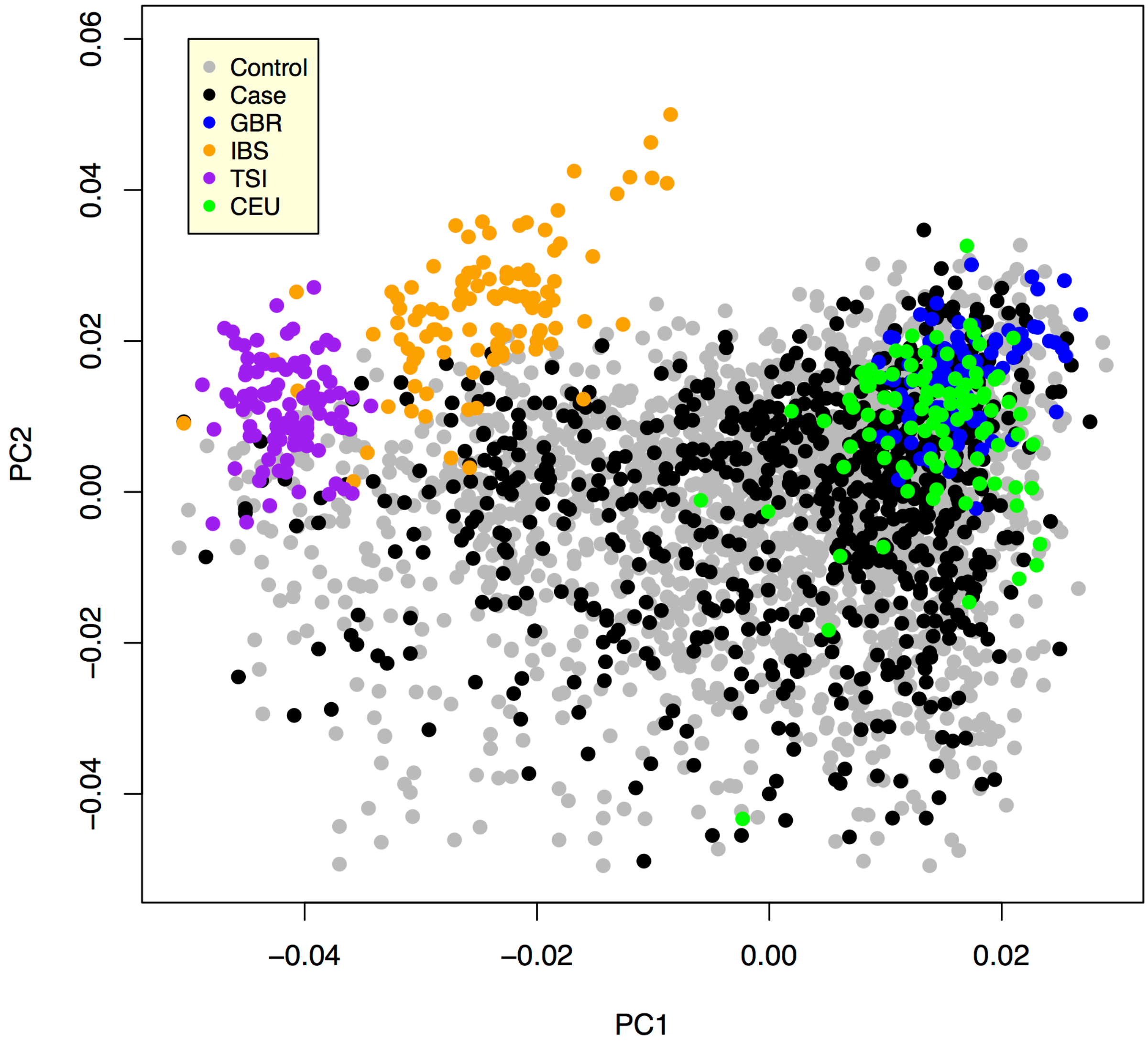

B. Population structures of the HH610 cohort.

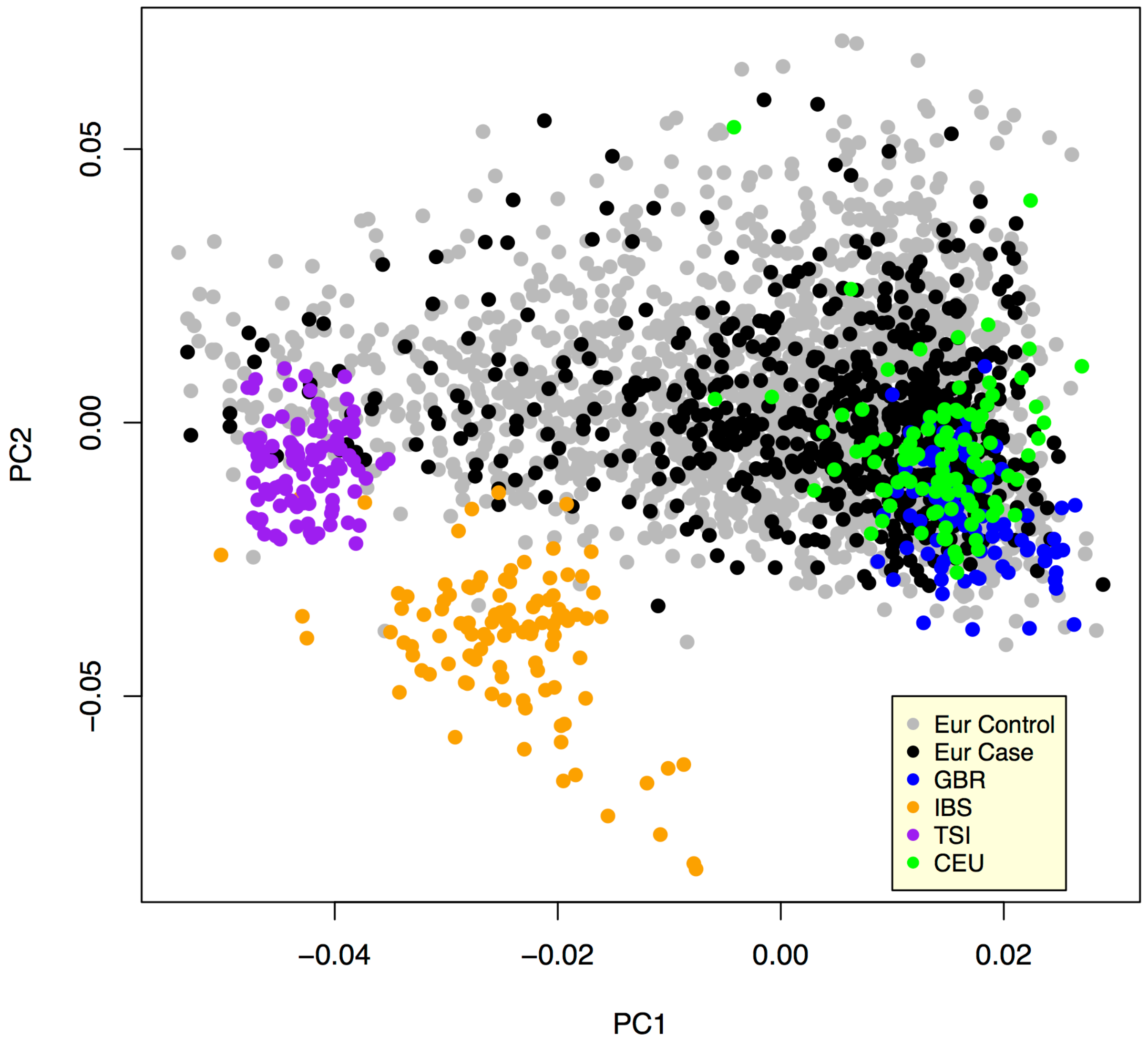

C. Population structures of the GSA cohort.

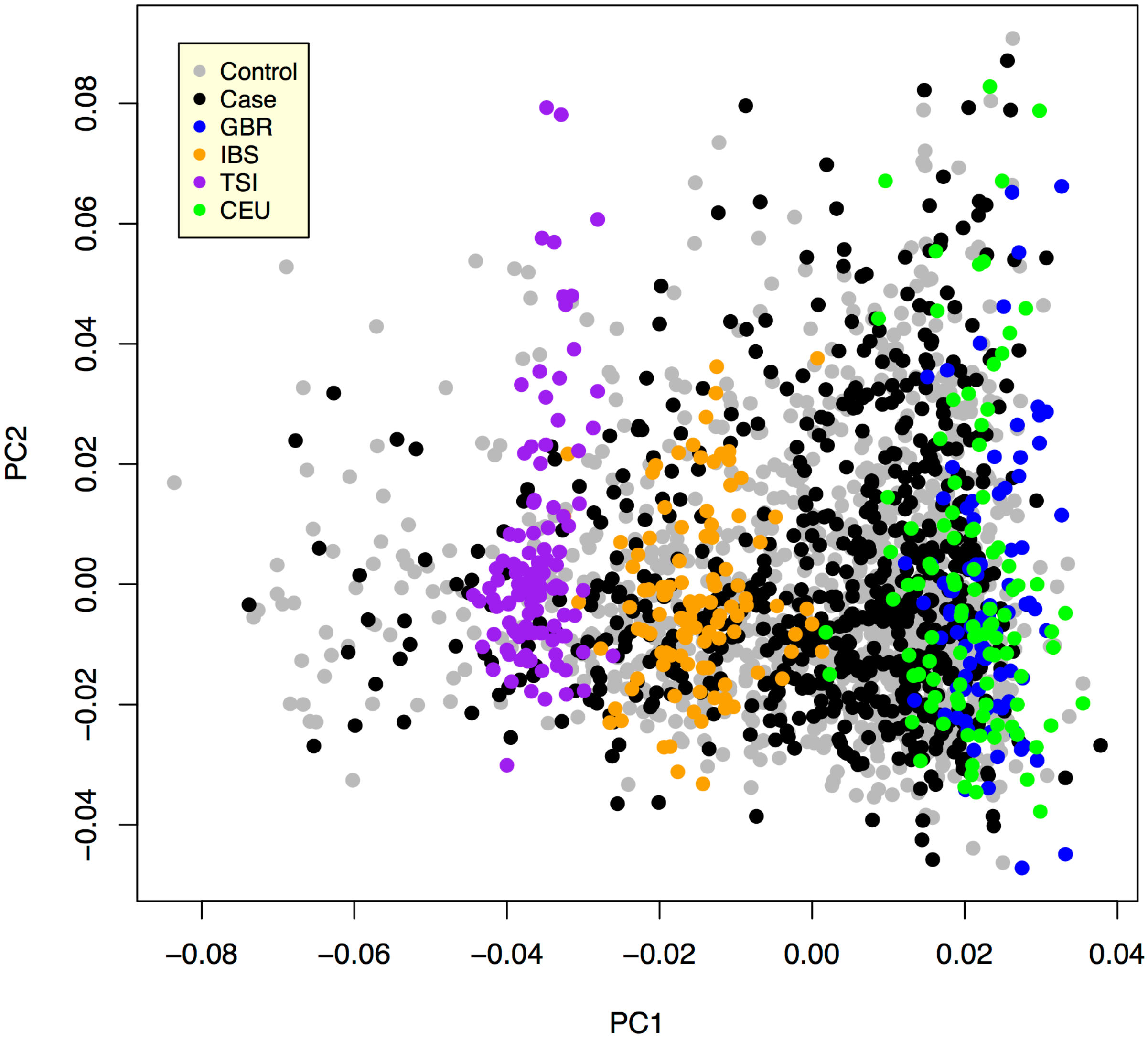

Supplement: Supplementary file 1 — Supplementary files [file 41398_2020_1064_MOESM1_ESM.pdf]
